# Invasive aspergillosis causing small bowel infarction in a patient of carcinoma breast undergoing chemotherapy

**DOI:** 10.1186/1477-3163-5-18

**Published:** 2006-06-06

**Authors:** Amit Chaudhary, Vinod Jain, Rama S Dwivedi, Samir Misra

**Affiliations:** 1 Department of Surgery, King George Medical University, Lucknow, India; 2 Department of Surgery, King George Medical University, Lucknow, India; 3 Department of Pediatrics, The Feinberg School of Medicine, Northwestern University, Chicago, USA

## Abstract

**Background:**

To report a 45 year old lady presenting with proximal jejunal gangrene due to invasive Aspergillosis. The patient was undergoing adjuvant chemotherapy for advance carcinoma of breast (Stage IV).

**Methods:**

The patient was referred to our surgical emergency for acute abdominal symptoms for 6 hours. Histopathology revealed bowel wall necrosis and vascular invasion by Aspergillus Fumigatus. Postoperative recovery was uneventful and the patient received Amphotericin-B (1 mg/kg/day) for invasive aspergillosis. Invasive pulmonary aspergillosis was confirmed by isolating Aspergillus Fumigatus from bronchoalveolar lavage and by a positive circulating galactomannan test (ELISA Assay).

**Results:**

Detailed history revealed dry cough and two episodes of haemoptesis for 2 weeks. Haemogram and counts revealed anemia and neutropenia. Plain X – ray of the abdomen showed multiple air fluid levels and ultrasound of the abdomen revealed distended bowel loops. On exploration small bowel was found to be gangrenous. The patient was successfully managed by supportive treatment and conventional intravenous Amphotericin-B for 2 weeks. The lady was discharged one week after completion of antifungal therapy and one month later she underwent toilet mastectomy. The lady came to follow up for 1 year and she is currently under hormone therapy.

**Conclusion:**

With the emergence of new and powerful immunosuppressive, anticancer drugs and potent antibiotics the survival of transplant and critically ill patients has remarkably increased but it has shown a significant rise in the incidence of invasive opportunistic fungal infections. We conclude hat the diagnosis of invasive gastrointestinal aspergillosis may be considered in a neutropenic patient with acute abdominal symptoms.

## Background

Despite the use of new and effective drugs, the disseminated invasive aspergillosis often remains lethal in neutropenic patients [1]. There is a great need to create high index of clinical investigation and develop better diagnostic tools to detect invasive aspergillosis before it causes irreversible damage. In the present study we report a 45 year old lady with severe neutropenia (400/cumm) presenting with acute abdomen. Invasive gastrointestinal aspergillosis leading to widespread proximal bowel infarction was found on exploration. The diagnosis was established by isolating Aspergillus fumigatus from bronchoalveolar lavage and by a positive circulating galactomannan test (ELISA). The patient received conventional intravenous amphotericin-B for 2 weeks.

## Methods

A 45 year old lady admitted in intensive care unit was referred to our surgical emergency unit for severe acute pain and distention of the abdomen for 6 hours. She was undergoing chemotherapy in clinical oncology department for locally advance carcinoma (Stage IV) of breast and four days back she was shifted to the intensive care unit when she developed severe neutropenia after fourth cycle CAF regimen (Cyclophosphamide 500 mg/m^2^, Adriamycin 50 mg/m^2 ^and 5-FU 500 mg/m^2^). There was no metastasis. Detailed history revealed that the patient had developed dry cough and fever 7 days back. The patient had absolute constipation. On examination the abdomen was distended and there was guarding and rebound tenderness. Hematocrit and counts revealed anemia (Hb 7 gm%) and neutropenia (400/cumm).

Plain radiograph of chest showed patchy infiltrates in both the lung fields suggestive of pneumonitis and X-ray abdomen showed distended bowel loops and multiple air fluid levels. There was no gas under the diaphragm. Ultrasound of the abdomen could not add much to the findings of plain X-ray and showed distended bowel loops and minimal interbowel fluid. Emergency exploratory laprotomy was performed under general anesthesia. About 30 cm of proximal jejunum 15 cm distal to duodeno-jejunal junction was found to be gangrenous. Primary resection and anastomosis was done and the specimen (resected bowel segment with adjoining mesentery) was sent for histopathology. After surgery the patient was shifted to postoperative ICU where she developed fever which was not responding to any antibiotic.

**Figure 1:**
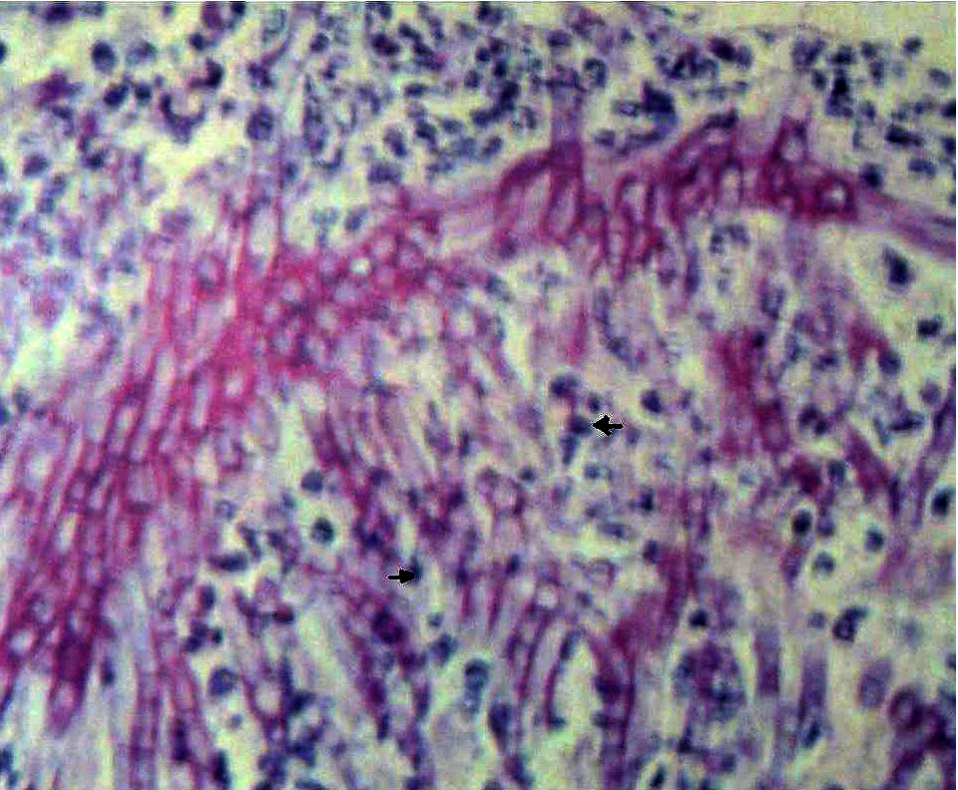
Histopathology analysis demonstrates vascular invasion by disseminated aspergillosis. (Arrows showing fungal forms).

On the basis of preoperative X-ray chest, neutropenia and high index of clinical suspicion broncho-alveolar lavage was performed which revealed pulmonary aspergillosis. Serum galactomannan test (ELISA) was also positive for Aspergillus. The diagnosis of invasive pulmonary aspergillosis was made and the patient was put on conventional intravenous Amphotericin B (1 mg/kg/day) for 2 weeks. Histopathology of the specimen was available on fourth postoperative day and revealed transmural ischemic necrosis of the bowel and widespread invasion and blockade of arterioles due to Aspergillus fungal forms (Fig. 1). The diagnosis of invasive gastrointestinal aspergillosis secondary to pulmonary Aspergillosis was made and antifungal treatment was continued. The patient responded to intravenous antifungal treatment and was discharged after 2 week of antifungal treatment. The lady underwent toilet mastectomy and was under follow up for 1 year. Postoperative chemotherapy was also given after improvements in the counts and presently she is under hormone therapy.

## Results and discussion

In our patient the diagnosis was made by exclusion and the decision for bronchi-alveolar lavage was taken which came out positive for aspergillosis. This evidence along with positive galactomannan test was enough to support the diagnosis of invasive Aspergillosis and intravenous Amphotericin B was started. The patient showed dramatic response and fever subsided on the next day. After recovery patient underwent modified radical mastectomy and postoperative chemotherapy and hormone therapy were also given. The patient was alive and well at the end of 1 year follow up.

Invasive gastrointestinal aspergillosis is an opportunistic infection that characteristically affects the immunocompromised host, resulting in high degree of morbidity and mortality [2]. The incidence of invasive aspergillosis has risen considerably during the past decade due to more intensive chemotherapy, organ transplantation, intensive care, and aggressive surgical interventions [3]. Extrapulmonary aspergillosis is rare but can involve gastrointestinal tract, heart, kidney, central nervous system, liver and pancreas etc [4]. Almost one-quarter of those with invasive pulmonary aspergillosis develop disseminated infection with Aspergillus sp. via hematogenous spread [2,5]. Among these patients the reported incidence of gastrointestinal involvement has been 41 – 47% [2,5,6].

Patients with bowel involvement are rarely diagnosed prior to autopsy because the typical symptoms of abdominal pain, obstruction and gastrointestinal bleeding are usually overshadowed by the septic manifestation of systemic infection. Wingrad et al [7] documented a case of aspergillosis as an unusual cause of perforated appendicitis in 1982. In our patient gastrointestinal invasion was secondary to hematogenous spread of fungal forms leading to ischemic necrosis of bowel. The diagnosis was made on the basis of high clinical suspicion followed by bronchoalveolar lavage and positive serum galactomannan test. This diagnosis should be considered when a neutropenic patients presents with abdominal pain and distention with fever.

There has been an increased understanding of the immunology of Aspergillus infection, paving the way to novel immune augmentation strategies in animal models that merit evaluation in phase I clinical trials [8]. Chest CT scan [8] and new techniques like PCR assays based on real time technologies that are able to quantify Aspergillus DNA are promising [9]. Considerable progress has been made in understanding the genetics of Aspergillus Fumigates and molecular techniques for manipulation of the fungus have been developed but still invasive Aspergillus's is associated with a high mortality rate ranging from 30% to 90% [10].

Repeated serum assays to detect Aspergillus antigenemia and meticulous search for other aspergillosis localizations like pulmonary, should continue until the diagnosis allows administration of early antifungal therapy. Though in our patient we used conventional antifungal treatment, combined antifungal therapy or treatment with amphotericin-B at high doses significantly reduces mortality as compared with the conventional treatment [11].

## Conclusion

Even with more effective and safer therapies, substantial progress in quelling aspergillosis may not occur until the early diagnostic tools are developed and incorporated into strategies to start earlier treatment. We suggest that a very high index of suspicion of invasive gastrointestinal aspergillosis may be maintained in immunocompromised patients presenting with acute abdominal symptoms.
